# Pain Experience During Rapid Maxillary Expansion: A Prospective Observational Study

**DOI:** 10.3390/children13030361

**Published:** 2026-03-02

**Authors:** Alberto De Stefani, Ayoub Boutarbouche, Martina Barone, Antonio Gracco, Giovanni Bruno

**Affiliations:** Department of Neuroscience, University of Padua, Via Giustiniani, 35121 Padova, Italy; alberto.destefani@unipd.it (A.D.S.);

**Keywords:** maxillary expansion, palatal expansion technique, pain, child, orthodontics

## Abstract

**Aim:** Rapid maxillary expansion (RME) is a widely used interceptive orthodontic procedure in pediatric patients that is often associated with discomfort and pain, particularly during the initial phase of treatment. The present study aims to analyze the intensity and temporal trend of pain perceived by pediatric patients undergoing rapid palatal expansion (RME) by evaluating the influence of factors such as age, sex, type of maxillary transverse deficit, palatal expander, and dental anchorage. **Materials and Methods:** A prospective observational study was conducted on 134 pediatric patients (mean age 8 years; range 6–14 years) diagnosed with transverse maxillary contraction. Patients were treated with tooth-borne Haas or Hyrax expanders. Pain perception was monitored daily using a specific questionnaire with answers based on the Wong–Baker FACES scale. Collected data were analyzed by using the ANOVA test (*p* < 0.05). **Results:** Pain was a common symptom, with greater intensity in the first days of treatment. The Hyrax expander was associated with significantly greater initial pain than the Haas one (*p* < 0.001). Tooth-borne expanders on the first permanent molars resulted in greater initial pain than anchorage on the second primary molars (*p* < 0.001). Patients with unilateral posterior crossbite and anterior crossbite reported higher average pain levels (*p* < 0.001). Age emerged as a significant modulator of pain (*p* < 0.001), while no significant differences were found based on sex (*p* = 0.287). **Conclusions:** Expander type, tooth-anchorage type, maxillary transverse contraction type, and patient age significantly influenced pain perception during pediatric RME. These results provided useful indications for the clinical management of the patient, for the choice of expander and anchorage type and for the timing of intervention.

## 1. Introduction

Rapid maxillary expansion (RME) is an orthodontic and orthopedic procedure frequently used to treat maxillary transverse deficiency in growing patients, which aims to increase the width of the upper arch by separating the mid-palatal suture [1,2]. RME can be obtained by using a device called rapid palatal expander (RPE), which is available in different types and conformations [2] and can include a dental, skeletal, or dento-skeletal anchorage. The activation of the RPE, which follows specific protocols, generates forces of a certain magnitude necessary to obtain the mid-palatal sutural disjunction and promote skeletal expansion of the maxillary jaw. Rapid maxillary expansion induces transverse widening of the maxilla primarily through the separation of the midpalatal suture, a growth site characterized by progressive morphological and histological changes during development. In early childhood, the midpalatal suture presents a relatively simple structure with limited interdigitation, allowing for more predictable orthopedic separation. As skeletal maturation progresses, increasing sutural interdigitation and surrounding circummaxillary resistance reduce skeletal responsiveness, leading to greater dentoalveolar effects and increased force transmission to teeth and supporting structures [3].

Despite the effectiveness of RME, adverse effects sometimes occur, such as pain, failure to open the intermaxillary suture and oral ulcerations [4]. Pain is a common symptom during RME, with a frequency that can exceed 90% in children treated with RME [5,6]. The etiology of orthodontic pain is considered as multifactorial, being influenced by individual variables such as age, gender, personal pain threshold and stress, as well as by the amount of applied forces [7,8]. The inflammatory process that occurs following the opening of the suture and the compression exerted on the periodontal ligament is thought to contribute significantly to the painful experience during RME [7].

Although pain during rapid maxillary expansion has been widely reported in the literature, its clinical modulation remains incompletely understood. Previous studies have mainly focused on the overall prevalence and temporal pattern of pain, or on comparisons between different expansion appliances or activation protocols [6,9]. However, evidence regarding the influence of specific clinical variables—such as the type of maxillary transverse deficiency, the type of expander, and the dental anchorage strategy—is still limited and often fragmented. While different appliance designs and anchorage teeth are known to generate distinct biomechanical force distributions, their impact on pain perception in pediatric patients has not been systematically investigated. Moreover, the role of the underlying transverse discrepancy as a potential modulator of pain remains poorly explored. Clarifying these aspects is clinically relevant, as pain perception may affect treatment compliance, parental acceptance, and the overall experience of interceptive orthodontic therapy. Therefore, a comprehensive evaluation of pain perception during RME that simultaneously considers demographic factors and key clinical variables is warranted. Tooth-borne rapid palatal expanders can be anchored either on the first permanent molars or on the second primary molars, depending on patient age, dentition stage, and clinical objectives. Anchorage on deciduous teeth has been proposed in early interceptive treatment to reduce undesirable effects on permanent teeth and to favor a more favorable force distribution, whereas anchorage on permanent molars is traditionally used in older children and adolescents.

Based on the available literature and clinical considerations, we hypothesized that pain perception during rapid maxillary expansion is influenced by both demographic factors and specific clinical variables, including expander design, tooth-anchorage type, and type of maxillary transverse deficiency. It was hypothesized that appliances generating higher localized forces and anchorage on permanent teeth would be associated with higher pain levels, especially during the early phase of treatment. The aim of the present prospective observational study was to analyze the intensity and temporal trend of pain perceived by pediatric patients undergoing RME, and to assess the influence of age, sex, type of maxillary transverse deficit, expander design, and tooth anchorage on pain perception.

## 2. Materials and Methods

### 2.1. Study Design and Questionnaire Used

A prospective observational study was conducted to evaluate pain perception and the occurrence of determined side effects during RME treatment in pediatric patients diagnosed with maxillary transverse contraction. The study protocol was approved by the local ethics committee (CET-ACEV: 543n/25). A structured and specifically created questionnaire was developed for the aim of the study by the Orthodontics research group of the School of Dentistry of the University of Padua. The first version of the questionnaire was ideated and drafted by one author for the purpose of the study. Then, this version was administered to 10 patients who had to start RME treatment. Data from this first phase of the study were not included in the final data analysis. Subsequently, the questionnaire, the answers collected and the participants’ feedback were examined by other three authors (who did not previously known questionnaire’s contents); the aspects assessed were the structure of the questionnaire, clarity and comprehensibility of the language used both in the questions and in the scale proposed to answer, effectiveness of the data collection methods and process, analyzability of the data obtained. Where necessary, questions and answers were reviewed and reformulated. Thus, the final version of the questionnaire was approved.

**Figure 1:**
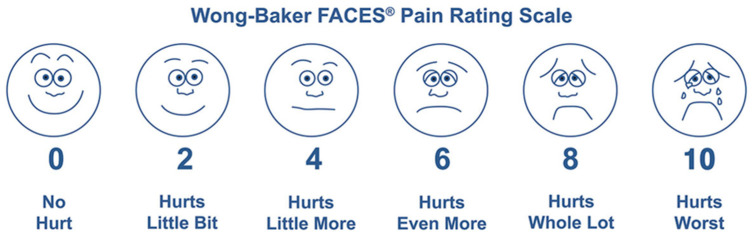
The Wong–Baker FACES^®^ Pain Rating Scale (from the Wong–Baker FACES^®^ Foundation).

The questionnaire consisted of the following parts:

1. Patient’s information—name, age, gender, type of maxillary transversal deficit (i.e., anterior crossbite, unilateral posterior crossbite, bilateral posterior crossbite, maxillary contraction without crossbite), type of expander used (Haas-type RPE vs. Hyrax-type RPE based on clinical indication), type of tooth anchorage used (bands on deciduous second molars vs. bands on permanent first molars), expansion protocol prescribed.
2. “Did you feel pain during activation?”—for each day the RPE was activated, the patients were asked to report the intensity of pain perceived. Patient were asked to use the Wong–Baker FACES^®^ Pain Rating Scale to define the perceived pain, validated in Italian for the pediatric age [10]. Figure 1 explain the structure and method of utilization of this scale.

Although the questionnaire was specifically developed for the purposes of this study, pain assessment was based on the Wong–Baker FACES Pain Rating Scale, a validated and widely used tool for pain evaluation in pediatric populations, including its validated Italian version. The remaining sections of the questionnaire were designed to collect clinical and demographic information relevant to the study objectives.

Since the patients were minors, written consent to use the collected data for research purposes was provided by their parents/legal guardians. They were also asked to assist the patients in completing the questionnaire.

Before enrollment, parents or legal guardians of all eligible patients received detailed oral and written information regarding the study aims, procedures, potential discomforts, and data management. Written informed consent for participation and for the use of anonymized data for research purposes was obtained from parents or legal guardians prior to the start of treatment. Participation was voluntary, and families were informed that they could withdraw from the study at any time without affecting the planned orthodontic treatment.

### 2.2. Study Sample

Patients were consecutively recruited among children presenting for orthodontic evaluation and treatment at the Orthodontic Clinic of the School of Dentistry, University of Padua. All treatments were performed within the framework of routine clinical care. The sample selection was performed according to specific and predefined inclusion and exclusion criteria. Patients aged 6 to 14 years, with mixed or early permanent dentition, diagnosed with maxillary transvers contraction (with or without unilateral or bilateral posterior crossbite, or anterior crossbite) were included in the study. All patients classified in the “anterior crossbite” group also presented an associated posterior crossbite.

All patients were treated with tooth-anchored RPE (type Haas or Hyrax), according to customized activation protocols. Patients with significant systemic diseases, genetic syndromes, neuromuscular disorders, previous facial trauma, congenital craniofacial anomalies, as well as individuals with previous orthodontic or maxillofacial surgery, were excluded. All Haas-type and Hyrax-type rapid palatal expanders were fabricated using conventional laboratory techniques with soldered components. No digitally designed or additively manufactured expanders were used in the study.

The choice of expander type (Haas or Hyrax) and tooth-anchorage configuration (bands on deciduous second molars or permanent first molars) was based on individual clinical indications and was not mutually exclusive. Both expander types were used with different anchorage configurations. Therefore, expander type and anchorage type were considered and analyzed as independent variables.

The expansion protocol was prescribed based on individual clinical indications and was therefore not fully standardized across all patients. Most of the sample followed a protocol of one activation per day, while a smaller proportion of patients were instructed to perform two activations per day or a combined protocol. The distribution of activation protocols is reported in Table 1. The mean duration of orthodontic treatment with rapid maxillary expansion was 11.5 months. The overall duration of the study, including patient recruitment, data collection, and follow-up, was 24 months.

### 2.3. Data Collection

On the day the RPE was cemented, a paper questionnaire was provided to the patient and their parents or legal guardians, together with standardized instructions on appliance activation, oral hygiene, and dietary precautions. Parents were specifically instructed to assist the child in completing the questionnaire daily, ensuring comprehension of the Wong–Baker FACES Pain Rating Scale, without influencing the child’s responses. Pain perception was recorded daily during each activation of the expander for the entire active expansion phase. Completed questionnaires were collected at follow-up visits and the data were anonymized and entered into a dedicated database (Excel, Microsoft Office 365, Microsoft, Redmond, WA, USA) for analysis. Only fully completed questionnaires were included in the final statistical evaluation.

### 2.4. Statistical Analysis

The statistical analysis was performed by the Department of Neuroscience of the University of Padua (Italy). As this was a prospective observational study based on a convenience sample derived from routine clinical practice, no a priori sample size calculation was performed. Descriptive statistics were calculated for all variables. Prior to inferential analysis, the distribution of quantitative variables was assessed for normality using the Shapiro–Wilk test. As pain scores showed an approximately normal distribution and considering the sample size, parametric statistical methods were considered appropriate. Mean pain scores were compared using analysis of variance (ANOVA) in relation to expander type (Haas vs. Hyrax), tooth-anchorage type (bands on deciduous second molars vs. permanent first molars), type of maxillary transverse deficit, patient age, and gender. Patient age was analyzed as an independent variable. The association between age and pain perception was explored by comparing pain scores across age groups using ANOVA.

When ANOVA revealed statistically significant differences among more than two groups, post hoc pairwise comparisons were performed using Tukey’s honestly significant difference (HSD) test. A *p*-value was calculated for each variable, and differences were considered statistically significant if *p* < 0.05.

## 3. Results

Considering the entire study population, pain perception was most pronounced during the initial phase of rapid maxillary expansion, with peak pain scores recorded within the first few days following appliance activation. Pain levels progressively decreased over time, reaching minimal or absent values by the end of the active expansion phase. No statistically significant differences in pain perception were observed between male and female patients throughout the observation period. Statistical analysis revealed several significant associations between the variables examined and pain perception.

### 3.1. Sociodemographic and Clinical Characteristics of the Study Sample

After the exclusion of cases with insufficient compliance (irregular activations or incorrectly completed diaries, n = 10) or failure to obtain informed consent (n = 5), the final study sample consisted of 134 pediatric patients. The sociodemographic characteristics included a mean age of 8.0 years (median: 8 years; interquartile range [IQR]: 7.0–8.5 years; range: 6–14 years), with 73 males (54.5%) and 61 females (45.5%). 47.0% (n = 63) of patients presented a maxillary contraction without crossbite (n = 63), 26.9% (n = 36) a unilateral posterior crossbite, 15.7% (n = 21) a bilateral posterior crossbite, and 10.4% (n = 14) an anterior crossbite. Patients were treated with a Haas-type expander in 27.6% (n = 37) of cases and with a Hyrax-type expander in 72.4% (n = 97) of cases. Dental anchorage was achieved with bands on deciduous second molars in 70.9% (n = 95) of cases and on permanent first molars in 29.1% (n = 39) of cases. Regarding the activation protocol, 78% of patients followed a regimen of 1 activation/day, 18% 2 activations/day, and 4% a combined protocol (2 activations/day for 10 days, followed by 1 activation/day). The mean number of total activations performed was 28.4 ± 12.6 (range 7–50) (Table 1).

### 3.2. Demographic Variables

Patient age emerged as a significant modulator of pain perception (ANOVA, *p* < 0.001). However, exploratory analysis did not reveal a clear linear relationship between age and pain intensity. Pain scores showed considerable interindividual variability across the age range, with overlapping values between younger and older patients. Exploratory descriptive analyses suggested that younger patients tended to report slightly lower pain levels during the initial phase of expansion, whereas older patients showed greater variability in pain perception. This observation was based on descriptive statistics and graphical representation and was not derived from a specific inferential statistical test. These findings suggest that age influences pain perception during RME, but the relationship is not strictly linear and may be modulated by additional clinical or individual factors.

### 3.3. Type of Maxillary Transversal Deficit

**Figure 2:**
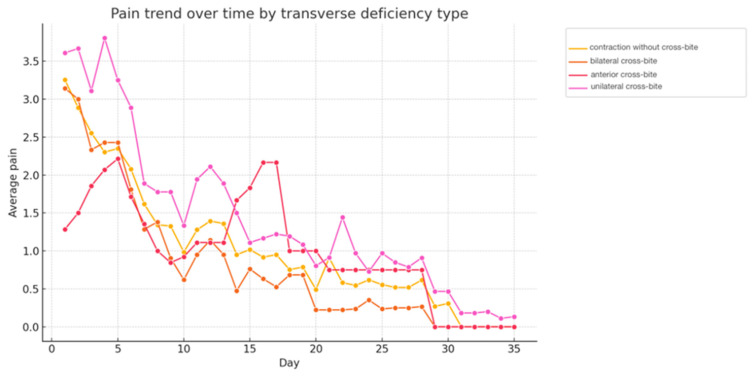
Pain trend over time based on type maxillary transverse deficiency type: maxillary contraction without crossbite, bilateral posterior crossbite, anterior crossbite, unilateral posterior crossbite.

Stratified analysis based on type of maxillary transversal deficit (Figure 2) showed that the “unilateral posterior crossbite” and “anterior crossbite” groups reported the highest average pain levels, especially during the first two weeks of treatment. Specifically, the “unilateral posterior crossbite” group showed a mean peak pain score of 3.8 around the fourth to fifth day, while the “anterior crossbite” group peaked at approximately 2.4–2.5 during the same period. Patients with “maxillary contraction without crossbite” (mean initial pain score ~3.1–3.2) and, to a lesser extent, those with “bilateral posterior crossbite” (mean initial pain score ~2.8–2.9) reported, on average, less pain, with the latter showing the lowest levels after the first week. Despite initial differences, pain progressively decreased for all groups, reaching minimal levels within 30 days. The differences observed between the types of maxillary transversal deficit were statistically significant (ANOVA, *p* < 0.001). Although ANOVA revealed a statistically significant overall difference in pain levels among the different types of maxillary transverse deficit, post hoc pairwise comparisons using Tukey’s HSD test did not show statistically significant differences between individual groups after correction for multiple comparisons. Therefore, the observed differences should be interpreted as descriptive trends rather than as evidence of specific intergroup differences.

### 3.4. Type of Rapid Palatal Expander

**Figure 3:**
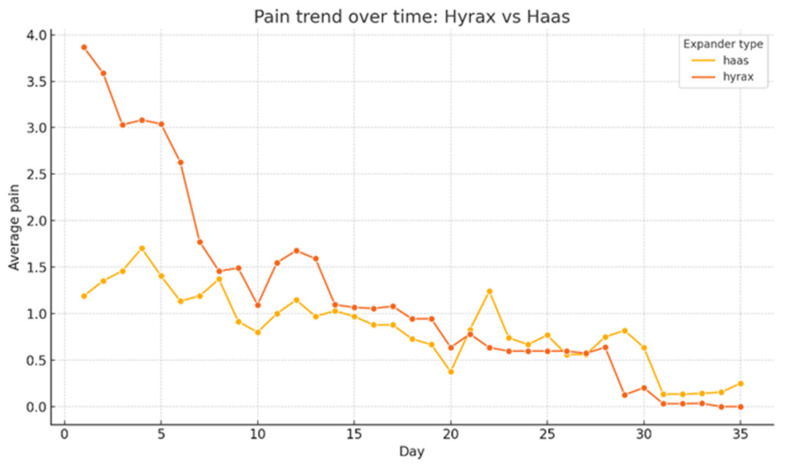
Pain trend over time based on type of RPE: Hyrax expander vs. Haas expander.

As illustrated in Figure 3, patients treated with the Hyrax expander reported higher average pain levels in the first few days of treatment, with a peak observed around the second or third day. Subsequently, pain intensity progressively decreased in both groups. After approximately 12 days, perceived pain levels in the two groups tended to converge, stabilizing at low or zero values towards the end of the observation period (35 days). The difference in pain trends between the two types of expander was statistically significant (ANOVA, *p* < 0.001). The influence of expander type on pain perception was evaluated independently from the tooth-anchorage configuration, which was analyzed as a separate variable.

### 3.5. Type of Tooth-Anchorage

**Figure 4:**
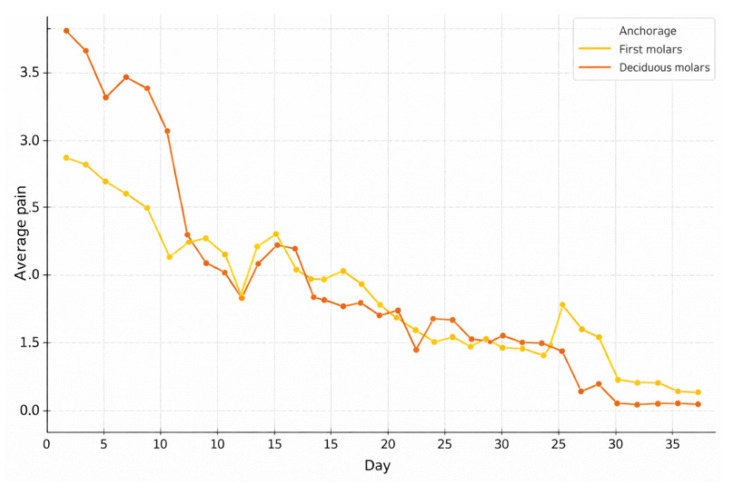
Pain trend over time based on type of tooth-anchorage: bands on deciduous second molars vs. bands on permanent first molars.

Comparing reported pain based on type of tooth anchorage (Figure 4), it was found that patients with bands on permanent first molars (“Bands on 6” group) reported higher mean pain levels than those with bands on deciduous second molars (“Bands on E” group), particularly during the first week of treatment. For the “Bands on 6” group, the mean pain intensity on day 1 was around 3.5, progressively decreasing to approximately 2.5 by day 5 and further to approximately 1.5 by day 7. For the “Bands on E” group, initial values were lower (day 1: ~2.3; day 5: ~1.5; day 7: ~1.0). Although both curves showed a general trend towards decreasing pain, from the second week onwards the differences between the two groups did not appear statistically significant, with both cohorts reaching minimal pain levels after approximately 30 days. The ANOVA statistical analysis indicated a statistically significant difference (*p* < 0.001) in the intensity of perceived pain in relation to the type of anchoring during the initial phase of treatment.

## 4. Discussions

The present study aimed to investigate the intensity and temporal trend of pain perceived by pediatric patients undergoing RME by evaluating the influence of different clinical and demographic factors. The present findings partially support the study hypothesis. In accordance with the initial assumptions, pain perception during rapid maxillary expansion was influenced by several clinical variables, particularly expander design and tooth-anchorage type, with higher pain levels observed during the early activation phase in patients treated with Hyrax-type expanders and with anchorage on permanent molars.

The emerged results indicate that pain is a frequently reported symptom, with a greater intensity in the first days of treatment, which tends to progressively decrease. This general observation is widely supported by the scientific literature, as also reported by the recent systematic review by Barone et al. [6], where it was highlighted that pain is a common effect of RME treatment that tends to decrease over time [8,9,10,11,12,13].

Regarding patient age, results identified this variable as a significant modulator of pain perception during RME (*p* < 0.001). However, the existing literature is not consistent on this point [6]. While Needleman et al. [14] did not report significant differences between age groups, Feldmann et al. [15] observed a positive correlation between age and pain perception on day 4 of RME, while Matos et al. [16] observed that younger children (7 years) tended to report less pain than older children (11 years). Thus, it is unclear if age can influence pain perception during RME [6,12]. Although the present study confirms the statistical significance of age on pain perception during RME, data presented do not specify the direction of this influence, i.e., whether pain increases or decreases with age within the study sample with a mean age of 8 years.

Regarding patient gender, authors did not find statistically significant differences in reported pain between males and females (*p* = 0.287). This finding is consistent with some of the available literature, which highlights a lack of general consensus and unanimous agreement [6,11,12]: some studies [14,15,16,17,18] reported no significant differences between gender, while others [9,15] suggested greater pain sensitivity or specific complaints in females. Therefore, data emerged by the present study contribute to supporting the hypothesis of poor or no influence of gender on pain perception during RME in pediatric population.

Statistical analysis of the present study also revealed that the type of maxillary transverse deficit significantly influenced pain perception (*p* < 0.001). In particular, patients with unilateral posterior crossbite and anterior crossbite reported the highest average pain levels. This observation suggests that the initial anatomical and occlusal configuration may influence the individual biomechanical response to maxillary expansion, as differences in sutural resistance and craniofacial constraints can affect stress distribution and displacement patterns in the maxillary complex [19,20].

Comparative analysis between Haas-type and Hyrax-type RPE revealed that patients treated with Hyrax-type RPE experienced significantly higher initial pain levels (*p* < 0.001). This finding is partially consistent with that reported by de Araújo et al. [9], who found significantly greater pain on the first day of therapy in patients treated with Hyrax-type RPE if compared to those treated with Haas-type RPE, although they concluded that, except on the first day, the type of appliance did not significantly influence pain perception. The hypothesis advanced by de Araújo et al. [9] and supported by Erverdi et al. [13] suggests that the design of the Haas-type RPE (made of acrylic resin pads in contact with the palate) may distribute the expansion forces more diffusely than the Hyrax-type RPE, which transmits the forces mainly to the periodontium of the anchorage teeth. The results of the present study seem to support this hypothesis with regard to the initial phase of RME treatment.

Another aspect investigated in this study was the type of tooth anchorage. It emerged that patients with bands cemented on the first permanent molars reported higher average pain levels, especially during the first week of RME treatment, compared with patients treated with anchorage on the second deciduous molars (*p* < 0.001). It is plausible that anchorage on permanent molars may increase periodontal loading during early activation, as tooth-borne expansion generates areas of periodontal ligament compression and inflammatory responses that are associated with pain perception. Moreover, early RME protocols anchored on deciduous teeth have been proposed to reduce undesirable effects on permanent molars, supporting the rationale that anchorage selection can modify the distribution of forces within the dentoalveolar unit [20,21]. A possible explanation for the lower pain levels observed in patients treated with anchorage on second primary molars may be related to differences in biomechanical response. Anchorage on deciduous teeth has been associated with greater dentoalveolar effects, including increased buccal inclination of the anchorage units, potentially resulting in a reduced magnitude of true skeletal expansion.

Although several studies have compared RME anchored on second primary molars with RME anchored on first permanent molars, these investigations have primarily focused on dentoskeletal, occlusal, or periodontal outcomes rather than patient-reported discomfort. For instance, randomized and multicentered trials have evaluated differences in dental arch response or transverse changes between deciduous- and permanent-tooth anchorage without including pain among the assessed variables, such as in the works by Ugolini et al. [22] on Haas-type expanders, or in CBCT-based analyses by Serafin et al. [23]. Likewise, studies examining buccal bone alterations around anchorage teeth, such as those by Lanteri et al. [24], have documented morphological responses but did not incorporate pain evaluation. At the same time, research specifically addressing discomfort during RME has primarily compared appliance designs, activation protocols, or adjunctive measures for pain reduction, rather than isolating the influence of the anchorage tooth type. Systematic reviews on RME-related pain, including that by Barone et al. [6], confirm that no available clinical trial has directly compared pain experience in patients undergoing the same expansion protocol with anchorage on deciduous versus permanent molars. Even the few studies reporting differences in pain between expanders anchored on primary teeth and other appliances (e.g., Quad Helix on permanent molars) do not permit a clear interpretation of the anchorage-related effect due to confounding biomechanical differences. Therefore, the findings of the present study contribute novel evidence by suggesting that the type of dental anchorage may modulate early pain response during RME and highlight the need for future research specifically designed to test this variable in isolation.

The results of this study, according to the existing literature, confirm that the clinician should adequately inform patients and their parents about the possibility of experiencing pain, especially in the first days of RME treatment. The choice of the type of RPE and tooth-anchorage can be useful considerations in therapeutic planning to minimize patient discomfort: according to the results of the present study, when clinically possible, Haas-type RPE and deciduous teeth anchorage should be preferred to reduce patient discomfort. Awareness of these factors may help clinicians optimize treatment planning and improve patient compliance, particularly in pediatric populations. When clinically feasible, the use of Haas-type expanders and anchorage on deciduous second molars may be considered to reduce early pain perception, especially in younger patients or in those with lower pain tolerance. Furthermore, patients presenting with unilateral posterior crossbite or anterior crossbite should be identified as potentially at higher risk of experiencing increased discomfort during the initial activation period.

From a clinical management perspective, the results support the importance of early interceptive treatment, as age was found to significantly modulate pain perception. Early intervention may not only improve skeletal outcomes but also reduce treatment-related discomfort, thereby enhancing acceptance of RME by both patients and parents.

Some limitations of the present study should be acknowledged. First, the observational nature of the study and the use of a convenience sample derived from routine clinical practice may have introduced a potential selection bias, which could limit the generalizability of the findings. However, consecutive recruitment and the application of strict inclusion and exclusion criteria were adopted to reduce heterogeneity. Second, pain perception was assessed using a self-reported scale, which, although validated for pediatric patients, remains inherently subjective and may be influenced by individual pain thresholds, emotional factors, and parental involvement. Additionally, the present study is the lack of full standardization of the expansion protocol, which was customized according to individual clinical indications. Different activation regimens may influence pain perception and therefore represent a potential confounding variable. Finally, although the sample size was relatively large, subgroup analyses may have been affected by unequal group distributions. These limitations should be considered when interpreting the results. Future randomized controlled studies with standardized protocols and objective outcome measures are warranted to further clarify the factors influencing pain perception during rapid maxillary expansion.

According to the recent literature [6], future research could explore more deeply the impact of the specific activation protocol on perceived pain. It would also be interesting to delve deeper into the relationship between age and pain during RME, as well as to further investigate the impact of different types of maxillary transverse deficit and type of tooth anchorage.

## 5. Conclusions

Pain perception during pediatric rapid maxillary expansion appears to be influenced by multiple clinical factors. Higher pain levels were generally observed during the initial activation phase, particularly in patients treated with tooth-borne expanders anchored on permanent molars and in those presenting more complex transverse maxillary deficits. Age influenced pain perception patterns, with older patients showing greater variability in reported pain. From a clinical perspective, these findings suggest that expander design and anchorage selection should be carefully considered when planning treatment, especially in patients at higher risk of discomfort. Enhanced patient counseling and pain management strategies may be warranted in the early activation phase, and patient age should be considered when timing intervention.

## Figures and Tables

**Table 1:** Sociodemographic and Clinical Characteristics of the Study Sample.

Age (Years)	Mean ± SD	8.0 ± 1.4
	Median (IQR)	8 (7.0–8.5)
	Range	6–14
Sex	Male	73 (54.5%)
	Female	61 (45.5%)
Type of transverse deficit	No crossbite	63 (47.0%)
	Unilateral posterior crossbite	36 (26.9%)
	Bilateral posterior crossbite	21 (15.7%)
	Anterior crossbite	14 (10.4%)
Type of expander	Haas	37 (27.6%)
	Hyrax	97 (72.4%)
Type of anchorage	Deciduous second molars	95 (70.9%)
	Permanent first molars	39 (29.1%)
Activation protocol	1 activation/day	105 (78%)
	2 activations/day	24 (18%)
	Combined protocol	5 (4%)

## Data Availability

The data presented in this study are available on request from the corresponding author. The data are not publicly available due to privacy and ethical reasons.
